# Social network and HIV risk behaviors in female sex workers: a systematic review

**DOI:** 10.1186/s12889-018-5944-1

**Published:** 2018-08-16

**Authors:** Zahra Jorjoran Shushtari, Seyed Ali Hosseini, Homeira Sajjadi, Yahya Salimi, Carl Latkin, Tom A. B. Snijders

**Affiliations:** 1 Social Determinants of Health Research Center, University of Social Welfare and Rehabilitation Sciences, P.O Box: 1985713834, Tehran, Iran; 2 Social welfare Management Research Center, University of Social Welfare and Rehabilitation Sciences, Tehran, Iran; 3 Social Development & Health Promotion Research Center, Kermanshah University of Medical Sciences, Kermanshah, Iran; 4 Department of Health, Behavior and Society, Department of Epidemiology, Johns Hopkins Bloomberg School of Public Health, Baltimore, USA; 5 Department of Sociology, University of Groningen, 9712 TG Groningen, Netherlands; 6 Nuffield College, University of Oxford, Oxford, OX1 1NF UK

**Keywords:** Social network, Social support, Systematic review, HIV risk behaviors, Female sex workers

## Abstract

**Background:**

Social network characteristics have an important role in understanding HIV transmission among female sex workers. The purpose of this systematic review was to summarize and critically appraise the existing studies on the social network characteristics and HIV risk behaviors among female sex workers.

**Method:**

A systematic review was performed using predefined eligibility criteria through searching electronic databases. Two independent reviewers assessed the methodological quality of studies.

**Results:**

Nineteen papers met the eligible review criteria. The synthesized evidence suggests that characteristics of social networks, especially functional characteristics such as social support and social capital, are important constructs for understanding the HIV risk behaviors.

**Conclusions:**

The findings of the present review enhance our understanding of the role of social network characteristics in HIV risk behaviors among female sex workers. However, the findings also highlighted a dearth of knowledge about the association of structural characteristics of social networks with HIV risk behaviors among female sex workers.

## Background

Global epidemiological surveillance data indicate that in 2015 about 36.7 million people were living with HIV. Of these, 2.1 million were new HIV infections in 2015 and about half of the newly HIV infected people were adolescent girls and young women [1, 2]. In the last decade, the incidence of HIV has decreased in several developed countries [3, 4]. However, the spread of HIV among high-risk groups such as men who have sex with men (MSM), female sex workers (FSWs), and people who inject drugs (PWID) is relatively high (44%), especially in developing countries [5, 6]. According to the World Health Organization report in 2015, about 74% of new HIV diagnoses were due to sexual transmission, 4% to injecting drug use, and for about 20% of the new diagnoses the transmission mode was reported to be unknown [7]. The UNAIDS 2016–2021 Strategy, with the aims to reach zero infections, absence of discrimination, and zero AIDS-related deaths, highlights the need for effective HIV prevention strategies for key populations [8].

HIV risk behaviors of injecting drug use and risky sexual behaviors are multidimensional and occur based on biological, individual, and structural factors. While individual attributes (including sex, age, education, occupation, and ethnicity) may influence a person’s attitudes, beliefs, and behaviors, various macro-level and social context characteristics can also contribute to engagement in, and continuation of, HIV risk behaviors [9].

A body of literature emphasizes the importance of social networks in HIV transmission and prevention [10–17]. As one of the first pieces of evidence on the key role of social networks, using data from 40 MSM with AIDS, Auerbach and colleagues reported in 1984 that HIV could be transmitted through sexual contacts and that having multiple sexual partners increases the probability of HIV transmission [18].

Since that time, numerous studies have shown that interpersonal interactions occurring in social networks, as well as network characteristics, are critical to understanding HIV risk behaviors and spread of infectious diseases more generally [11–16, 19, 20]. It has also been shown that social network approaches may be helpful for HIV intervention and to allocate resources more efficiently for preventive strategies [21].

A social network is a set of ties among people who have some common interests or interactions [22]. Family, friends, neighbors, coworkers, and sex or drug partners may be members of the social network that influence HIV-related behaviors. A network can affect the members’ behaviors and health outcomes; this may be based on structural network characteristics such as size (the number of members of the network), density (the extent to which network members are connected to each other), degree (an individual’s number of direct ties), betweenness (frequency of ties with which an individual is on the shortest path connecting pairs of others in the network), centrality (extent to which an individual has a central position in the network), and homogeneity (similarity between network members) [9, 23–26]. Rothenberg et al., in a study of in sexual transmission of syphilis among teenagers in rural Georgia, showed that structural characteristics of the network position of individuals such as degree, betweenness, and information centrality facilitated the transmission of syphilis. Participants with syphilis had a higher degree (on average 7.4 sexual partners) compared to those without syphilis (2.4 sexual partners). Similarly, the participants with syphilis had an average betweenness of 4.1, which was higher than the average betweenness of 1.7 for those without syphilis. This network parameter indicates that participants with syphilis were more central in the network than those without syphilis [27].

According to the literature, larger networks provide more opportunities for exposure to a variety of risks, health information, and practices affecting health behaviors and outcomes of network members [9, 28]. Furthermore, HIV risk behaviors often occur in the context of a dense social network where risk behaviors are normalized, and information can pass easily and frequently between individuals [9, 29]. Some studies have shown that larger sexual networks are associated with increased reporting of unsafe sex among MSM and of syringe sharing among PWID [25, 28, 30]. Also, social networks may influence risk and health behaviors through various psychosocial mechanisms and tie characteristics such as frequency of contact, tie duration, social influence, social norms, close contacts, provision of social support, and social capital [31]. One study among PWID in India showed that the PWID who had more than 10 PWID in their drug network were 1.65 times (95% CI: 1.12 to 2.42) more likely to have shared a syringe at the last injection compared to those who had 0 or 1 member in their networks. These authors found also that participants with the largest injection drug network size were 31% (95% CI for relative proportion: 0.53 to 0.90) less likely to be virally suppressed compared to those with the smallest network size [32]. A study among 385 male migrants in China showed that condom use norms of the core network were significantly associated with the participants’ condom use. Participants with one or more network members who always used condoms were 12 times (AOR: 11.9, 95% CI: 2.4–59.0) more likely to consistently use condoms than participants with no such alters in their sex work networks [33].

While some studies have investigated the association of social network structure and function with HIV risk behaviors in teenagers, HIV-at-risk women, PWID, and MSM [29, 34–37], there are few studies that have systematically reviewed the existing literature about this association for FSWs, who are an important group at risk and also hard to reach in many countries [5]. In addition, they may be a bridge group for HIV transmission to the general population [38, 39].

Some systematic reviews have assessed networks and health. Perkins et al. focused, in a systematic review in 2013, on how social network structure and influential individuals within a network may reinforce health outcomes and behaviors in low- and middle-income countries [40]. They found network composition, position, and structure to be related to health outcomes and behaviors. Although these authors considered HIV transmission as one of the health outcomes in the general population, they did not consider HIV risk behaviors specifically among FSWs. Qiao et al. in 2014 conducted a systematic review on the association between social support and HIV-related risk behaviors among groups such as drug users, MSM, adolescents, people living with HIV/AIDS, and FSWs [41]. This review found 5 studies on FSWs and confirmed the role of social support in reducing HIV risk behaviors.

Despite these interesting findings, these reviews have produced limited information on the role of social networks for HIV risk behaviors among FSWs. Therefore, a systematic review with a focus on functional and structural characteristics of social networks may be valuable to support future interventions for HIV prevention among FSWs. The purpose of the present review was to review and summarize existing quantitative and qualitative studies about network structure and function of FSWs and their association with HIV risk behaviors.

## Methods

This review was conducted in December 2016 using electronic search in databases including Web of Science, PubMed/Medline, Scopus, Ovid; the publishers Springer and Science Direct; and the key journal of AIDS and Behavior. Google Scholar was also searched. The search period was from 1990 to 2016. Additional articles were identified from manual reference checks of relevant studies. A sensitive search strategy was used to retrieve relevant studies. The Medical Subject Headings (MeSH) controlled vocabulary system was used to define the keywords. The combination of keywords used for PubMed was (Social network OR social support OR social capital) AND (AIDS OR HIV OR human immunodeficiency virus) AND (drug use OR risky behavior OR risky sexual behavior) AND (female sex worker OR sex worker OR prostitute), and equivalent specifications were used for the other databases. This review has not registered a protocol. For data management, all retrieved studies were imported into EndNote (version X).

### Eligibility criteria

Inclusion criteria for the studies, in agreement with the PECOT structure (Population, Exposure, Comparison, Outcome, Time), were observational and qualitative studies of which the studied population consisted of female sex workers (FSWs), assessed the association between structural characteristics (e.g., size, density, stability, etc.) or functional characteristics (e.g., social support, social capital) of the social network and HIV risk behaviors, were peer reviewed, and published prior to December 2016. Only primary studies published in English were included. Studies that did not provide information on the pre-specified PECOT items were excluded, regardless of their methodological quality.

### Study selection and data extraction

Two reviewers appraised the papers independently in two steps: title/abstract and full-text review. In the first step, after excluding duplicate papers, if reviewers had disagreements to consider a title or abstract as relevant, discussions were held until consensus was reached. The two independent reviewers examined the abstracts of the remaining articles based on the inclusion criteria. Methodological quality was assessed using forms of the Critical Appraisal Skills Program (CASP) [42]. The CASP checklists cover methodological rigor, the validity of results, and the relevance of results to practice [42]. Each question in the critical appraisal was scored as no or insufficient quality (score 0), medium quality (score 1), or sufficient quality (score 2). Quality scores were calculated from the individual items in the checklists. The mean quality score was calculated as the quality sum score of each article divided by the number of items in the critical appraisal forms. Based on the study design, specific CASP critical appraisal forms were used. The quality scores were not used for including the studies. The reviewers were not blinded to the names of authors and journals. Discrepancies between reviewers were resolved by the judgment of a third person and consensus. The intra-class correlation coefficient (ICC) was used, with its 95% confidence interval, to assess the agreement between reviewers. The multi-faceted synthesis method of meta-study was used to integrate methods and findings into descriptive summaries [43]. According to this method, we assessed methodological aspect of the primary studies such as sampling, data collection, and research design. The similarities and differences between the results of the studies were also assessed to categorize them and formulate conclusions. The data for the included articles were summarized as Author (s), location, sample size, study design, and main findings.

## Results

**Fig. 1:**
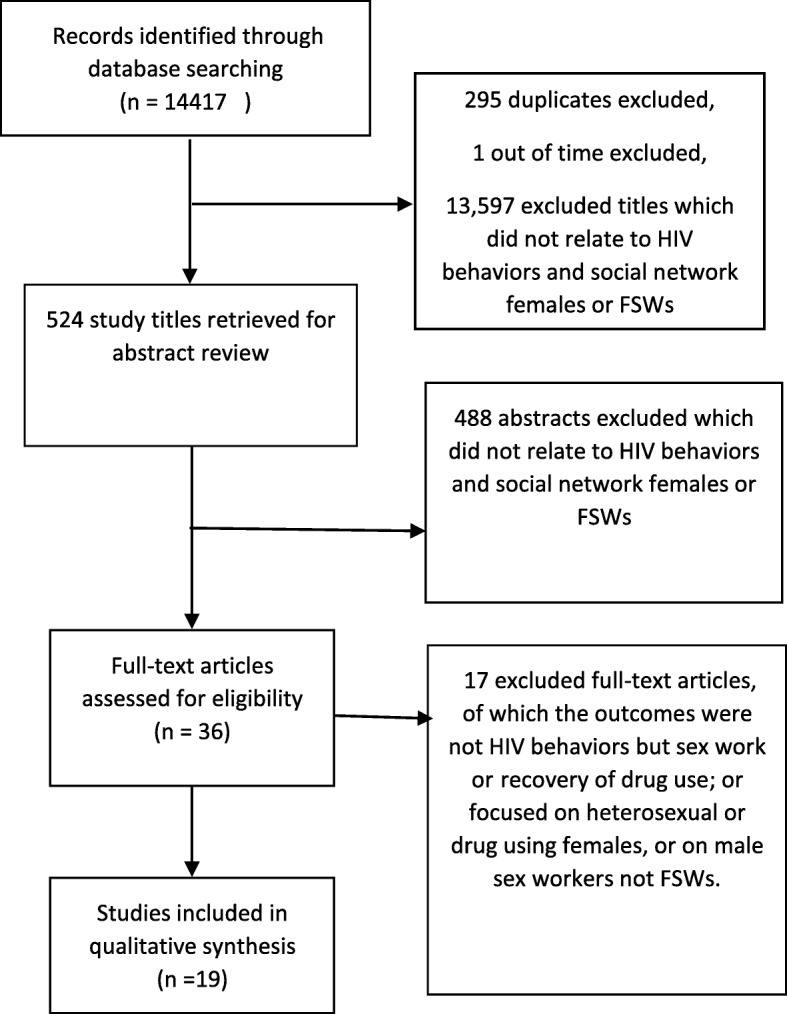
Flow diagram of screening and selection studies

Figure 1 shows, based on the PRISMA guidelines [44], details of the process from the initial search and screening to final study inclusion. The search criteria identified 14,417 papers in the primary search, of which 524 were listed for the abstract review. Based on the inclusion criteria, 36 papers remained for the full text review. Nine of the included studies focused on heterosexual drug using females but not FSWs [34, 45–52], and one study reported the role of social networks as mediators of sexual abuse and HIV risk among drug using women [53]. The outcome variables of four studies were sex work or drug users’ recovery efforts and viral suppression but not HIV risk behaviors [54–57]. The studied population in three studies consisted of male sex workers rather than females [58–60]. Finally, 19 studies met the eligibility criteria (Fig. 1).

We retained thirteen quantitative and six qualitative studies. Twelve of the studies were conducted in Asia [38, 61–70], three in the USA [71–73], one in Swaziland, and the others were conducted in other countries (Table 1). Of the quantitative studies, ten were cross-sectional [61–63], and one was a longitudinal study that appears to have been reported in three related published papers [71–73]. There was strong agreement between the two reviewers concerning the methodological quality, ICC = 0.95 (95% CI: 0.87 to 0.98). The included studies were of high quality with respect to research design rigor. Mean scores for qualities of research methodology for each study are presented in Table 1.

**Table 1:** Summary of reviewed related quantitative and qualitative studies (*N* = 19)

Author (s), location	Sample size	Study design	Functional/Tie characteristics^a^	Structural characteristics^b^	Quality score^c^	Main findings
Rothenberg et al., USA [72]	595 persons at high risk for HIV, 133 FSWs, 129 their paying partners, 47 non-paying partners, 200 injecting drug users, 41 their sex partners.	Longitudinal study	–	Network size density stability	1.62	There was a negative correlation between the total network size and the stability index. This correlation was significant for sexual relations (*r* = − 0.28, *P* < 0.01) and social (*r* = − 0.26, P < 0.01) networks, but not for drug-using network (*r* = − 0.13, *P* > 0.10)
Klovdahl et al., USA [71]	111 persons at high risk for infectious disease including HIV, 48 FSWs, 35 their partners, 24 injecting drug users, 4 their sex partners	Longitudinal study	-	network size density Reachability	1.38	The median of network size was 11.7 (range 0, 54). Of the relationships involving risk behaviors, about 25% were reported to be sexual (anal & non-anal), 23% involved in drug sharing (non-?A3B2 show $132#?>needle), and 6% needle-sharing. The participants were found to be highly interconnected. The adjusted density of network connection was 0.046. Reachability was 1. Of the three observed HIV positive persons, one was in the connected region and was a paying partner of FSWs. Based on the graph-theoretic terms, they can reach their three personal associates directly in one step and the entire core of the connected region in six steps.
Woodhouse et al., USA [73]	595 persons at high risk for HIV, 133 FSWs,129 their paying partners, 47 non-paying partners, 200 injecting drug users, 41 their sex partners	Longitudinal study	–	Network position Network component	1.62	The 595 respondents identified 5162 people to which they belonged as network members. More than 70% of respondents perceived themselves to be at low risk for HIV infection. Network analytic methods showed 147 separate connected components. Eight of the 19 HIV-positive persons in the network were placed in smaller components remote from the largest connected component.
Dandona et al., India [63]	6648 FSWs	Cross- sectional Study	Social support	–	1.9	Inconsistent condom use with clients was associated with low social support (OR = 2.60; 95% CI = 2.17, 3.12) and not participating in FSW support group (OR = 2.02; 95% CI = 1.50, 2.70).
Li et al., China [62]	318 FSWs	Cross- sectional Study	Social support	–	1.7	Perceived gatekeeper support was positively associated with consistent condom use with clients (OR = 1.80, 95% CI = 1.08, 3.03).
Yang et al., China [61]	454 establishment-based FSWs	Cross- sectional Study	Social support	–	1.9	Perceived gatekeeper support was associated with condom communication (with clients: Adjusted OR = 1.99 (95% CI = 1.35, 2.95); with stable partners: Adjusted OR = 1.44 (95% CI = 1.02, 2.06)), and consistent condom use (with clients: Adjusted OR = 1.45 (95% CI = 1.07, 1.96); with stable partners: Adjusted OR = 1.5 (95% CI = 1.09, 2.08)). However, it was not associated with proper use of condoms.
Kerrigan et al., Dominican Republic [74]	288 FSWs	Cross- sectional Study	Intimacy	–	1.7	After controlling for socio demographic characteristics of participants, low perceived intimacy with the most recent regular paying partner (OR = 7.20 (95% CI = 3.49, 14.83)) was significantly associated with condom use prevention in multivariate analysis.
Reisner et al., Boston, Massachusetts [79]	11 Transgender male-to-female sex workers	Qualitative-Mixed method Study	Social support	–	1.4	Social networks play an especially vital role in the lives of transgender women, who face ongoing stigma and discrimination in negotiating their identities, and remain socioeconomically disadvantaged. These factors may affect access to clinical care and/or disclosure of behavioral HIV risks to medical and mental health providers. Participants overwhelmingly discussed support groups or other avenues of networking with other transgender women as an area of interest for HIV prevention.
Tucker et al., South china [65]	34 low-income FSWs, 28 Health outreach	Qualitative Study	Frequency of contact Trust	–	1.6	Sex workers Laoxiang (hometown social network contact, women who migrated from the same region) influenced condom use through several mechanisms such as promoting wholesale condom purchasing, mediating condom use with clients, and providing options for managing clients who refused condom use. Outreach members observed that sex workers accompanied by their Laoxiang were often more willing to accept STI/HIV testing and trust local sexual health services.
Chen et al., Shanghai, China [67]	21 Female entertainment workers (16 from large venues and 5 from small venues)	Qualitative Study	Type of contact Network role Social support	–	1.8	There were several personal networks in both large and small entertainment establishments in Shanghai, China that based on unique conditions were efficient for the diffusion of safer sex messages. Madams, who act as intermediaries between FSWs and clients, had a main role in FSWs’ social networks, but did not act as information disseminators and support the FSWs for condom use due to a conflict of interest between safer sex and maximizing profits. Messages about safer sex and condom use appeared to be more easily disseminated when the information could be present from people working at different levels in the venues.
Lau et al., China [75]	158 FSW -Non Injecting Drug Users (FSW-NIDUs) and 218 FSW-IDUs	Cross- sectional Study	Social support	–	1.5	According to multivariate analysis results, lack of social support was significantly associated with inconsistent condom use during commercial sex among FSW-IDUs but not among the FSW-NIDUs (Adjusted OR = 2.93, 95% CI = 1.28–6.70).
Ye et al., China [38]	504 FSWs	Cross- sectional Study	Social support	–	1.7	After controlling for socio-demographic characteristics in multivariate analyses, an environmental-structural support variable (which was measured by a scale composed of as “enabling and reinforcing factors supporting condom use in the establishment including perceived level of safe sex information exchange among employees (FSWs); support from the establishment owner (gatekeeper) about the important of condom use during commercial sexual services; accessibility of condoms in the establishment for condom use”) was the most significant positive predictor of consistent condom use (OR, 3.96; CI, 2.52–6.22) among FSWs and their regular paying partners.
Urada et al., Philippines [64]	143Female entertainment workers trading sex	Cross- sectional Study	Frequency of contact Social influence Informational support	–	1.8	Among participants, those who had less frequent contact with their managers (Adjusted OR = 0.46 (95% CI = 0.27, 0.78)) and were not following their co-workers’ advice to use condoms (Adjusted OR = 0.13 (95% CI = 0.04, 0.44)), used condoms less consistently.
Fonner et al., Swaziland [77]	324 FSWs	Cross- sectional Study	Social capital	–	1.8	Social cohesion among female sex workers was associated with consistent condom use with all partners in the past week (Adjusted OR = 2.25 (95% CI = 1.30, 3.90)). Social participation was associated with always using condoms with non-paying partners (Adjusted OR = 1.99 (95% CI = 1.13, 3.51)).
Januraga et al., Indonesia [66]	34 FSWs	Qualitative Study	Social capital Reciprocity Solidarity	–	1.7	Newcomer sex workers often experienced intensely competitive working environments fueled by economic competition. This competition reduced opportunities for positive social networks and social learning about HIV prevention. The lack of social networks and social capital between FSWs undermined peer trust and solidarity, both of which are essential to promote consistent condom use. Therefore, these increase their HIV risk.
Hao et al., China [68]	63 older FSWs (28 street-based and 35 venue-based sex workers) and 53 pimps, roadside salon and hotel owners	Qualitative Study	Social support	size, density, frequency of contact, role of network members	1.5	Based on the functional and structural characteristics of FSWs’ social network as size, density, frequency of contact, role of network members and social support, family networks (children and husbands) and workplace networks (peers, clients, pimps, and owners) differently influenced (promoted or deterred) FSWs’ condom use.
Gu et al., China [69]	200 FSWs who are injection drug users	Cross- sectional Study	Social support	–	1.7	In final multivariate model, after adjusting for socio-demographic variables, perceived social support from family members and friends (OR = 0.39, 95 %CI = 0.12–0.44) had significant association with condom use.
Cruz Serena, Uganda [80]	FSWs who live in slums and brothel	Qualitative Study-ethnography	Intimacy Social support Social capital	–	1.6	Social network through intimacy, trust, social support and social capital provides a basis for managing daily risk related living in brothel and HIV risk behaviors related sex work. Social relationships especially with other FSWs and their social support in brothel can help the FSWs when they encounter insecurity with money, physical harm, and illness.
Yang et al., China [70]	1916 female entertainment sex workers	Repeated Cross- sectional Study	Social support	–	1.5	In final multivariate model, after adjusting for other individual and social covariates, only peer support for condom use remains a significant and independent correlate of consistent condom use in sex with a non-stable partner (OR = 1.08, *P* < 0.01). Peer support can promote a normative environment supportive of safe sex and reinforce risk reduction behavior.

^a^Functional characteristics include functions of interaction between network members, e.g., social support and social capital. Tie characteristics are characteristics of ties or interactions between people in a network including frequency of contact, duration of tie, intimacy between network members, etc. In this review some of the included studies considered some of these functional and also some of these tie characteristics

^b^A network in which people interact with each other has structural characteristics including size (the number of members in a network), density (to what extent people are connected to each other), centrality, homogeneity (to what extent people are similar to each other in a network), etc.

^c^Mean quality score was calculated as quality score of each article divided by number of items in related critical appraisal forms. Attainable range score 0–26 for the longitudinal studies and 0–20 for the cross-sectional and qualitative studies

### Measurement of HIV risk behaviors

The HIV risk behaviors outcome was assessed differently across the studies. Although most studies considered only condom use as an HIV risk behavior outcome [38, 63, 66, 68, 70, 74–76], some studies considered outcomes of drug use, number of injections, and needle sharing [72, 73]. All studies relied on self-reports, including condom use or injection or non-injection drug use. A variety of single-item and multiple-item composite outcome measures were used to assess HIV risk behaviors. The measurement of risky sexual behaviors also was heterogeneous. Some studies assessed condom use only in sexual relationships with clients or paying partners [74, 75], but other studies measured condom use with clients and with other regular partners or non-paying partners (lover, boyfriend, and husband) [61, 63, 64, 77]. In the qualitative studies, HIV risk behaviors were assessed based on reported knowledge and experience of FSWs with respect to sexual practices (such as condom use and condom negotiation) and sexually transmitted infections [66]. For example, one qualitative study asked the participants “when you came to a brothel for the first time, did you know about condoms?”, “Have you ever used condoms?”, “Why did you accept him without using condoms?” [66].

### Measurement of the social network

#### Name generator and name interpreter

Measurement of personal networks usually proceeds in two steps: name generator and name interpreter [78]. A name generator is a question asking participants to nominate network members according to a specific criterion. Name interpreters are questions about each network member mentioned, and about the relationship between the respondent and the network member. Most of the studies asked respondents about social support and other interactions with others in specific roles such as boyfriend, lover, client, peer or co-worker, and relatives, without making an inventory of the network by a name generator [61, 63, 67, 79]. Only one of the studies, a longitudinal study [71], used a name generator to nominate network members. Participants were asked to name their network members, defined as those with whom they have “close personal contact” in the six months before the interview. Close personal contact in this study could have been any type of relationship but the study emphasized listed relationships by which HIV can be transmitted, such as injecting drug use and sexual contacts [71]. The network interpreter asked questions about the relationship between the participants and the named network members, and about demographic and locating characteristics.

#### Measures of functional characteristics

Most of the studies did not consider network structure and only measured functional characteristics such as social support or social capital [38, 63, 65, 70, 75–77]. These studies assessed the emotional, instrumental, and information support received from network members, especially peers and gatekeepers. Two studies evaluated mutual aid, trust, and solidarity, interpreted as social capital [66, 77]. Social support in some of the studies was measured by one question [61–63]. Three studies measured social support by multiple (four to eight) items [38, 70, 76]. One study measured social support by one dichotomous question, “yes” or “no” [75]. Only one study used an established scale for measuring social support, adapted from the Norbeck social support questionnaire [64]. Social capital in one study was measured by an established scale developed in Brazil [77]. In one study, social capital was explored using qualitative methods including open-ended, semi-structured interviews [66]. Most of the studies reported only Cronbach’s alpha, varying between 0.56 to 0.97, as a reliability index of the measures. One study reported both validity and reliability indices [74]. One study did not report any validity or reliability indices [75].

### Measures of structural characteristics

Structural characteristics were measured in diverse ways. Three articles came from one longitudinal study in the USA of the sociocentric (sociometric, or whole social network) network, and provided information about the direct and indirect relationships in the network [71–73]. This study assessed structural characteristics such as network size, size of the connected component, position, density, centrality, and stability of the network over the time. One qualitative study investigated the sociocentric network using in-depth interviews and focus group discussions, and assessed network size, density, composition, and contact frequency [68]. Two studies only considered the frequency of contact and intimacy relationships (operationalized as trust, affection, and love) among FSWs, FSWs and their clients (or partners or lovers), or FSWs and their gatekeepers [64, 74]. One of the studies used the “Norbeck Questionnaire” for measuring frequency of contact [64], whereas in the other study frequency of contact and intimacy relationships was measured directly [74]. The characteristics and main findings of the included studies are summarized in Table 1.

### Social network and HIV risk behavior

Most studies focused on the role of social support and social capital in condom use with clients or other sexual partners [61–63, 65, 77]. Seven of the cross-sectional studies found that social support from network members, especially gatekeepers (manager or pimp) and peers, was significantly associated with condom use of FSWs [38, 61–63, 68, 70, 75].

FSWs with high perceived social support were more likely to use condoms than those who perceived low social support [38, 61–63, 68, 70, 75]. One of the cross-sectional studies, conducted in China, reported that FSWs who perceived social support from gatekeepers were more likely to consistently use condoms compared to those with low social support (OR = 1.80, 95% CI = 1.08, 3.03). Also, Yang et al. in their study among 454 establishment-based FSWs in China similarly reported that perceived social support from gatekeepers was associated with condom communication with clients (Adjusted OR = 1.99 (95% CI = 1.35, 2.95) and stable partners (Adjusted OR = 1.44 (95% CI = 1.02, 2.06) [61]. However, one of the qualitative studies, also in China, found contradictory findings regarding the social support from gatekeepers and its role in condom use among FSWs [67]. The authors found that gatekeepers, due to the conflict of interest between safer sex and FSWs’ health on the one hand and financial benefits, on the other hand, did not disseminate information about condom use and safe sex among FSWs and did not support FSWs for condom use [67]. Two qualitative studies in China also reported similar results as the quantitative studies about the positive role of social support from network members in condom use among FSWs [65, 68]. One of these qualitative studies in China found that individuals in the Laoxiang network, which was the hometown social network, referring to women who migrated from the same region and live together, provided peer support to each other (‘Laoxiang sisters’). This was important for their health behaviors such as obtaining and using condoms, managing clients who refuse condom use, promoting health care behaviors including STI/HIV testing, and reducing the effects of anti-prostitution campaigns. The Laoxiang sisters through informational support facilitated access to condom use. Female sex workers who lived in the same hometown consulted each other about condom use and condom negotiation with clients. They also provided informational support for HIV testing and helped each other to decrease distrust of medical organizations for HIV testing [65].

The role of social capital in FSWs’ condom use was assessed in three studies [66, 77, 80]. One showed that participants who had a network with high levels of social cohesion, as a social capital construct, consistently used condoms with all partners 2.25 times more often than those who reported low levels of social cohesion (Adjusted OR = 2.25; 95% CI = 1.30, 3.90) [77]. In addition, people who had high levels of social participation consistently used condoms with non-paying partners nearly twice as often as those who had low levels of social participation (Adjusted OR = 1.99; 95% CI = 1.13, 3.51) [77]. The qualitative studies reported that the lack of social capital among FSWs decreased peer trust and cohesion, which were found to be essential for consistent condom use [66, 81].

In the present systematic review study, only 32% of the studies (*n* = 6) considered the relationship between social network structural characteristics and HIV risk behaviors. Three quantitative articles with a longitudinal study design, all based on the same study [71–73], and one qualitative study [68], focused on structural characteristics of social networks such as network size, density, stability, position analysis, and their role for HIV transmission and HIV risk behaviors. The longitudinal articles highlighted the key role of the network configuration in the dynamics of HIV transmission [71–73]. The authors studied the network configuration and changes over time in network characteristics of stability, position, centrality, and size of the main connected component [71–73]. The median network size was 11.7 (range 0–54) [71]. Participants were found to be highly interconnected, and the adjusted density of the network was 0.04 [71]. One of the articles reported a significant negative correlation between network size and stability of the sexual network (*r* = − 0.28, *p* < 0.01) and stability of the social network (*r* = − 0.26, *p* < 0.01) [72]. Results showed that the HIV positive clients of FSWs who are in the connected component of the network could, on average, infect three of their ties directly in one step, and the entire core of the connected region indirectly in six steps [71, 72]. These authors showed that social network structures could provide pathways for HIV spreading among the FSWs [71–73]. One qualitative study in China among 63 FSWs who were at least 35 years old explored the influence of structural, functional, and relational characteristics of their social networks on safe sex practices (condom use) [68]. This qualitative study found that there were two major types of networks influencing FSWs’ condom use, family networks and workplace networks. These two types of social networks and their structural and functional characteristics influenced condom use among older FSWs. However, actual condom use among most of the FSWs was low because their decision to use condoms was often determined by clients and their desire to make money to support their families. The FSWs who lived with peers or co-workers had a larger network size, more frequent contacts with FSW peers, and higher levels of HIV/STI-related informational, tangible, and emotional support and supportive norms regarding condom use, including acceptance of condom use. By contrast, older FSWs who lived with husband or children were relatively isolated or had smaller network size and also received from their peers little HIV/STI-related informational, tangible, and emotional support about condom use [68]. Two studies only assessed the association of frequency of contact and intimacy with condom use [64, 74]. These studies showed that FSWs who had low perceived intimacy and less frequent contact with their gatekeeper (or manager or pimp) and peers were less likely to use condoms. Also, a cross-sectional study showed that significant associations with inconsistent condom by FSWs were social network factors including daily contact with manager or gatekeeper (Adjusted OR = 0.46; 95% CI = 0.27, 0.78), following co-worker’s advice for condom use (Adjusted OR = 0.13; 95% CI = 0.04, 0.44), and having medical personnel as an informational source for HIV prevention (Adjusted OR = 0.29; 95% CI = 0.11, 0.77) [64].

## Discussion

In recent decades many countries, especially developing countries, continue to experience a steady increase in the numbers of people living with HIV/AIDS. About 74% of HIV transmission is related to sexual contacts [2, 7]. Female sex workers are among the most important groups who are at risk of HIV. Given the challenges of prevention of HIV transmission, it is important to focus on HIV risk behaviors among this high-risk population. The present systematic review considered studies of social networks of FSWs and, in particular, the functional and structural characteristics of the networks and their associations with HIV risk behaviors.

Only 19 studies were identified over the period between 1990 and 2016, none of which focused exclusively on the association of social network characteristics and HIV risk behaviors of FSWs. Most of the relevant studies focused only on the role of social support and social capital in condom use among FSWs [38, 61–63, 67, 77]. Only four of the included studies assessed the structural characteristics of FSWs’ social networks and their association with HIV risk behaviors [68, 71–73].

Most of the studies did not include a network name generator and an associated network interpreter to obtain relevant information about the content of the ego-alter relationships [61, 63, 67, 79]. Although these studies did provide some information about exchanges of social support and other resources between egos and alters in these networks, using roles such as regular partner, client, peer or co-worker, and relative, we do not have sufficient information about quality and quantity of these relationships and of the exchanges of social support and other resources.

Consistent with past research by Qiao [41], we found that social support and social capital as functional characteristics of social networks were significantly associated with HIV risk behaviors, especially condom use. The broader focus of the current review compared to that by Qiao led to finding a greater number of studies (19 vs. 5), more information about name generators and name interpreters, and more extensive results in terms of functional and especially structural characteristic of FSWs’ social networks and their association with HIV risk behaviors. Given the findings of our review, peers and gatekeepers appear to have a key role in the social network of FSWs and can affect their condom use [38, 61–63, 68, 70, 75]. Most of the included studies showed that social support, trust, intimacy, and solidarity with peers and gatekeeper are positively associated with condom use among FSWs [38, 61–63, 68, 70, 75]. When frequency of contact, trust, and social support are high, peers and co-workers can be effective in educating FSWs about prevention of HIV risk behaviors [74, 82]. Peers and co-workers can facilitate the dissemination of messages about protective health behaviors, teach each other, and improve each other’s power of negotiation with clients or sexual partners about condom use. A lack of social support, by contrast, may increase social isolation and reduce the motivation for learning safe sex behaviors from peers, disclosing HIV status, and insisting on protective behaviors, such as condom use [64, 66, 75, 83]. Further, some of the included studies showed that gatekeepers (pimps or establishment owners) who manage sex workers have an important role in condom use of FSWs and their clients [61, 62]. Gatekeepers can provide a supportive environment for safe sex behaviors via their educational messages, determine condom use rules in the workplace, and enforce client condoms use. However, one of the studies found that gatekeepers may be a barrier for promoting safe sex among FSW due to the conflict of interest between financial benefits and the FSW’s health [67]. Our findings suggest that interventions for promoting condom use among FSWs should consider the role of gatekeepers and peers, and of contextual factors such as contact frequency, trust, intimacy, and social support in the social network of FSWs. Interventions should aim to improve trustful and supportive relationships with peers. Furthermore, condom use messages should be designed to be easily disseminated through the peers or gatekeepers in the FSWs’ social network. Valente et al. in their study on the association between social networks and contraceptive use among women in Cameroon found that the women’s contraceptive use was associated with their perception of their network partners’ support for contraception and with their network partners’ encouragement for contraceptive use [20].

These findings can be used to develop and implement relevant behavior change interventions. Potential intervention strategies include training programs for two types of network members. The first approach would be to train peers to diffuse safe sex information and skills in condom use negotiations with clients and regular partners [84]. A second approach would be to train gatekeepers in creating supportive norms of condom use, by providing educational programs and condom use skill trainings, enacting a mandatory condom use rule with penalties for the rule’s violation, promoting condom use negotiations with clients, and by providing FSWs with free condoms [85]. For example, a peer education program intervention for HIV prevention among FSWs in Bangladesh, which assessed the effects of social support provided by peer educators to FSWs, found that the FSWs who received more informational support or emotional support from their peer educators reported a higher rate of using condoms, more self-efficacy, as well as lower self-reported STI symptoms at follow-up [84].

In addition, the review findings show that structural characteristics of FSWs’ social networks are associated with HIV risk behaviors [71, 72]. However, only four of the relevant studies assessed the structural characteristics of FSWs’ social networks and their association with HIV risk behaviors [68, 71–73]. The results of the longitudinal articles showed that the network size, density, network position, centrality, and stability might have a role in HIV transmission [71–73]. According to this study, a large dense network is more likely to have members who share HIV risk behaviors with each other. This finding supports the perspective that dense networks can provide more pathways along which behaviors, as well as diseases, may flow [9]. Also, based on the findings, FSWs and their clients who are HIV positive and are connected directly or indirectly in the sexual network cannot only transmit the infection to each other but also act as a bridge for HIV transmission to other networks and populations. The authors suggested that positions of persons in networks with different structural characteristics may have a different effect on the rate of HIV transmission. In a sexual network with low density and centrality, HIV positive persons who are clients and occupy a peripheral or isolated position may be less likely to transmit HIV to the network. By contrast, a densely connected network structure in which HIV positive persons are highly central may facilitate transmission of HIV [72]. These findings also show that changes of the network might be crucial for understanding the dynamics of HIV transmission.

Only one of the included qualitative studies focused on both the structural and functional characteristics of FSWs’ social networks and their association with HIV risk behaviors [68]. This study showed that structural and functional characteristics of FSWs’ different social networks (family and workplace network) might influence condom use of FSWs, be it negatively or positively. The authors found that support from peers and pimps in FSWs’ work network may promote their condom use. By contrast, family members, especially the presence of children in the social network may exacerbate the need to make more money and have a negative influence on FSWs’ safe sex behavior [68]. This study also found that FSWs who had a larger work network, with higher density, and more frequent and supportive contacts with peers, had safer sex than those who were isolated or had smaller networks with low density and fewer contacts with peers. Despite these interesting findings, the qualitative results of this study do not provide strong evidence about the association of social network characteristics with HIV risk behaviors.

This review highlights the heterogeneity of approaches to measurement used to assess social network characteristics of FSWs. This heterogeneity is related to different study designs, different definitions of the concepts of social support and social capital, and the use of a variety of instruments for measuring social networks and their complex properties. Some limitations of the measures in the related studies were the lack of a theoretical or conceptual framework with respect to potential effects of social network characteristics on HIV risk behaviors of FSWs, insufficient measurement of social network characteristics, measurement of HIV risk behaviors only by self-reports, recall bias, and a lack of information regarding reliability and validity of instruments.

The present systematic review hopes to provide insights into understanding the social network characteristics of FSWs, especially the role of structural characteristics of these networks for HIV risk behaviors. This review provides evidence about the positive association of social support with condom use among FSWs. This information may help researchers and public health planners to develop HIV prevention intervention for FSWs. However, due to the heterogeneity of approaches to define and measure social support, we could not combine the results and generalize across all included studies. Despite the findings regarding the role of network structure for HIV transmission and risk behaviors, this evidence, based on just one quantitative and one qualitative study, is not sufficient to provide a reliable conclusion about the role of structural characteristics of FWS’s social network on HIV risk behaviors. Therefore, to address the question regarding which structural characteristics of FWS’s social network may affect HIV risk behaviors, it is necessary to conduct additional research.

Limitations of the present systematic review are the following. First, non-English and unpublished studies were not included. Second, the search strategy used was broad, but still some articles may have been missed. Third, collection of demographic variables and social network characteristics was not consistent across the studies; because of this diversity, the findings could not be combined in a meta-analysis. Fourth, most of the studies included were cross-sectional, so that it is impossible to draw any causal inferences between social network characteristics and HIV risk behaviors.

Despite these limitations, the findings of the present systematic review have important suggestions for future studies and interventions. First, future studies need to pay attention to methodological and measurement issues. For example, future studies should be guided by theoretical frameworks to examine the mechanisms expressing how social network characteristics may affect HIV-related risk behaviors of FSWs. In addition, we suggest that future social network studies use types of network inventory (name generator and interpreter) that are frequently used in personal network studies [78], to provide sufficient information about quality and quantity of relationships between ego and alters in a network. The number of existing longitudinal studies was very limited, consisting of only one study which had three published articles. Longitudinal data are necessary to provide stronger information on causal relationships between social networks and HIV-related risk behaviors.

Second, further studies should consider structural characteristics of FSWs’ social network.

Social networks with different structural characteristics may have a different effect on HIV risk behaviors and HIV transmission among FSWs. For example, FSWs in a sexual network with high density, where more clients know each other and where the centrality of HIV-positive clients is high, may be more affected by HIV than FSWs who are engaged in a sexual network with a low density in which HIV-positive clients occupy a peripheral or isolated position [72].

Information about structural characteristics of FSWs’ social networks such as density, degree, betweenness, and centrality that can facilitate diffusion of behaviors, information, disease transmission, is necessary to develop an effective HIV prevention intervention among FSWs. Such information will aid in the design of network interventions among FSWs and help policymakers to allocate resources for HIV prevention programs.

Third, to provide a more complete picture of FSW’s social networks, future studies should examine both structural and functional characteristics of social networks and their association with HIV risk behaviors among FSWs to provide sufficient information about the structural and psychosocial mechanisms through which the relationships between network members may affect health-related behaviors and outcomes of FSWs. Only one qualitative study in China considered both structural and functional characteristics of social networks and their association with HIV risk behaviors among FSWs.

Fourth, further studies should focus on the quantity and quality of ties among peers as well as ties with gatekeepers and sexual partners (clients and regular partners), and the dynamics of these relationships within the social networks of the target population. The HIV risk behaviors of FSWs may be embedded in power dynamics between FSWs, gatekeepers, sexual partners, and peers. For example, FSWs with smaller size peer network or low frequency of contact with peers who can support safe sex behaviors of FSWs may be more dependent on their partners and maintain the emotional intimacy with their partners even through unsafe sex [66, 86].

## Conclusions

The present review provides evidence of the complexity of the network of FSWs, composed of different sub-networks or network sources such as family, peers or co-workers, gatekeepers, clients, and regular partners. Different network structures may have different effects on HIV risk behaviors of FSWs. According to the findings of the present review, social support and social capital as functions of social networks are important constructs for understanding FSWs’ HIV risk behaviors, especially condom use. The findings highlighted a lack of knowledge about the association between structural characteristics of the social networks of FSWs and their HIV risk behaviors. The results obtained by the included studies are not sufficient to clarify the mechanisms according to which social network structures of FSWs affect their HIV risk behaviors. Understanding such mechanisms of action, and improved knowledge of social network characteristics of FSWs more generally, may lead to the development and implementation of more effective intervention programs for prevention of HIV transmission. We recommend policymakers and practitioners to design, implement, and evaluate new and more systematic and rigorous network approaches in prevention and harm reduction intervention that target HIV risk behaviors among FSWs.
